# Publishing gold standard science

**DOI:** 10.1172/JCI201281

**Published:** 2025-11-03

**Authors:** Elizabeth M. McNally, Sarah Jackson, Corinne L. Williams, Oliver Eickelberg

The US federal administration recently unveiled a plan titled Restoring Gold Standard Science to ensure that “federally funded research is transparent, rigorous, and impactful, to ultimately improve the reliability of scientific results” (1). The NIH adopted these guidelines on August 22, 2025, publishing a modified plan as Leading in Gold Standard Science (2). These plans have followed a number of current and past efforts to improve the reliability of biomedical research, with considerable attention given to preclinical studies, especially those using animal and cell models. The preclinical emphasis is aimed at promoting successful translation of basic research findings to human disease and enhancing the development of effective medications. As we enter this period of focusing on the validity of NIH-supported scientific research, journal policies and practices will continue to play an important role in meeting these needs. We encourage the NIH to recognize publishers’ role in conveying research results with transparency and accuracy. In this respect, the *Journal of Clinical Investigation* (*JCI*) and *JCI Insight* will continue to rely on peer review that, by design, includes disagreement and dissent — hallmarks of scientific progress. In addition, members of the journals’ professional staff deploy a number of additional data integrity checks on manuscripts and take steps to promote the accessibility of underlying data. We encourage the NIH to evaluate the effectiveness of past efforts at enhancing reproducibility so that policy shifts are better informed and rapidly implemented.

A 2012 commentary by Begley and Ellis on the quality of published preclinical data drew wide attention to the problem of irreproducibility in cancer research (3). The authors described efforts to validate 53 influential oncology studies, finding that only six could be reliably reproduced. They hypothesized that this lack of reproducibility contributes to the high rate of failed oncology clinical trials. To address these challenges, Begley and Ellis called for higher standards in experimental design, including proper blinding, randomization, and statistical rigor, as well as greater transparency in methods and reporting. In response, the NIH devised its 2014 Rigor and Reproducibility (R&R) framework, requiring grant applications to explicitly address the scientific premise, methodological rigor, consideration of biological variables including sex, and inclusion of information on the authentication of key resources (NOT-OD-15-103, NOT-OD-15-102). These elements were incorporated as application review criteria. Grant reviewers were trained to ensure these criteria were addressed during the review process. Alongside these efforts, the NIH also expanded requirements for NIH-supported trainees to include a mandated curriculum on R&R during fellowship and career development programs. Collectively, these initiatives elevated expectations for researchers with the goal of enhancing reproducible science for NIH-supported research.

Over the past decade, the NIH has also progressively strengthened its expectations around data availability and sharing. Data sharing principles were honed by the NIH *Genomic Data Sharing (GDS) Policy*, governing large-scale genomic data, and then ultimately broadened across digital data types. In 2015–2016, the NIH began to emphasize the need for open access availability of publicly funded science. The NIH *Policy for Data Management and Sharing*, first published in 2020, applies to all NIH-funded or NIH-conducted research generating scientific data and requires investigators to include a data management and sharing plan with their grant applications. Compliance with the data management and sharing plan is a condition of an NIH award and must be addressed in annual progress reports. Data sharing and open access policies are essential, and they contribute to the R&R framework.

## Journal standards have evolved in parallel

In parallel with establishing the R&R framework in 2014, the NIH engaged journals and scientific societies to elevate standards for reporting preclinical research (4). *Principles and Guidelines for Reporting Preclinical Research* emphasized the need to report on randomization, masking, sample-size estimation, and data handling (5). Although not uniformly used, there are now standardized frameworks for reporting animal experiments (6, 7). At the *JCI* and *JCI Insight*, we have focused on improving reporting on sample size and statistical analysis, enhancing reporting of reagents used, clarifying our standards for immuno blot data, and setting policies mandating access to high-throughput sequencing datasets (8). In 2024, the *JCI* and *JCI Insight* began requiring authors to publish all values underlying graphs presented in figures and behind any reported means. We also began mandating publication of raw immunoblot data, which the JCI has required authors to submit for review since 2012. In 2025, our staff began manual quality control measures of high-throughput sequencing and proteomic datasets prior to acceptance. We believe that journals have a substantial role in enabling data management and sharing by requiring data deposition in publicly accessible data repositories and/or housing information on the journals’ databases. By aligning grant expectations with editorial practices, the NIH together with journals have driven a cultural shift in which methodological consistency and transparency are recognized as fundamental to the credibility of preclinical science.

## The publisher’s role in reporting R&R

While the R&R framework outlines design and execution of experiments, detailed reporting of methods and results must be held to the same high standards. Beyond standardized reporting guidelines, journals are using additional methods to improve data integrity prior to publication. While data integrity screens vary widely across different journals, they mostly employ a combination of computational and manual approaches. In 2016, we introduced manual screening of images in manuscripts, followed by artificial intelligence–based (AI-based) image screening in 2021 (9). Importantly, AI screening of data integrity prior to publication requires extensive human input, even with the aid of software tools to screen for image duplication and plagiarism, as these generate a number of false-positive or -negative results. Current data integrity scans are further augmented by detailed manual examination of other data including immunoblots. Data presentation standards used by the *JCI* and *JCI Insight* include the requirement to present data distribution, as this is an essential component to assess effect size and variability. All individual data values used in plots and graphs must be provided in the manuscript at first submission so that our editors and peer reviewers can adequately evaluate any submission. We individually verify at the time of submission that large datasets are appropriately deposited in public databases or, if applicable, there is a compelling reason why such a deposit has not been performed (such as protecting participant identity). Thus, while each of these processes is rather time consuming and costly, the *JCI* and *JCI Insight* routinely employ these data integrity screens, recognizing these steps as essential to ensuring the credibility of the published work and the journals alike.

## The role of journal peer review and editors in R&R

Gold standard science, as noted by NIH Director Jayanta Bhattacharya, relies on robust education of scientists and rigorous peer review (2). We concur. We also believe dissent and disagreement are alive and well in scientific inquiry and publishing. The *JCI* was among the very first journals to formally and systematically use peer review, beginning in 1942 (10). The peer review process was implemented and designed to expose scientific disagreement and should be free from ideological contamination. Indeed, it is rare to find manuscript evaluations in which all reviewers are all aligned. Even less commonly is there full agreement among authors and reviewers. As editors, it is our mandate to adjudicate these differences. At the *JCI* and *JCI Insight*, our editorial boards include a wide range of active scientists, who use their own experiences in this process. Submitted manuscripts with diverging reviewer opinions are discussed in editorial board meetings with a large number of attending experts, after which the editors conclude with a decision.

Do we always get it right? Probably not, but we weigh each of the opinions carefully, and to their credit, authors often incorporate these ideas into the description of a study’s limitations. We are convinced that this process ultimately improves the scientific content and R&R of any paper. The scientific method relies on reproducibility. Lack of reproducibility derives from flawed concepts and/or limitations of experimental methods and models, and far less commonly from outright fraud or fabrication. The fractal edge of new scientific knowledge is driven by a core component of discord, reflected in peer review and the editorial process. While some journals choose to publish reviewer comments to authors or published manuscripts, we believe that the ability for reviewers to speak frankly under masked and unpublished peer review also holds value.

## What is known about the effectiveness of the R&R framework?

**Figure 1:**
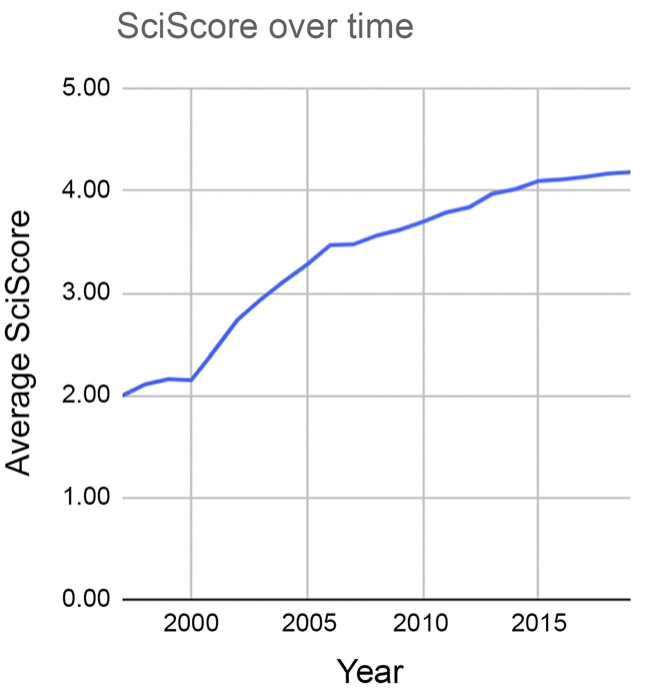
Average SciScore, an index of rigor and transparency, over time. The largest increase was realized between 2000 and 2012, prior to the implementation of the NIH R&R framework in 2014. From 2014 to 2019, increases in the average SciScore were more modest. Reproduced from ref. 12.

Given the time and resources dedicated to implementing the R&R framework, we welcome more efforts to assess the effectiveness of this framework. Tools such as the SciScore Rigor and Transparency Index (RTI) are used to analyze specific components of published papers vital to the R&R framework (11, 12). Although imperfect and complicated by non-uniformity in reporting formats, data reveal a doubling of the SciScore (from ~2 to ~4) between the years 2000 and 2012, a timeline that predates the NIH framework on R&R (Figure 1). After introduction of the R&R framework, the SciScore improved comparatively little (<5%) between 2014 and 2019. Thus, the greatest improvements in SciScore were realized independent of the R&R framework.

We hypothesize that in the US, underfunding also threatens R&R. We note that the doubling of SciScore values between 2000 and 2012 could reflect NIH budget doubling, which began in the late 1990s. During this time, there were many remarkable advances in scientific methodologies that included more reliable and consistent molecular biology and biochemical methods, technical advances in microscopy, enhanced computational abilities, next-generation sequencing, and the evolution of large, publicly accessible databases. It would be valuable to know what accounted for this apparent improvement in R&R and whether improved reporting of R&R contributed to — or whether funding, education, or other factors enhanced — R&R. Critically, we also do not know whether increased attention to R&R has yielded better preclinical translation. NIH policy changes that affect grant applications, peer review, training, and data reporting are costly and may be cost effective. However, as scientists who are stewards of taxpayer dollars in biomedical research, we encourage better accountability to show that these requirements represent a good investment. Performative policies serve little public good.

## Conclusions

**Figure 2:**
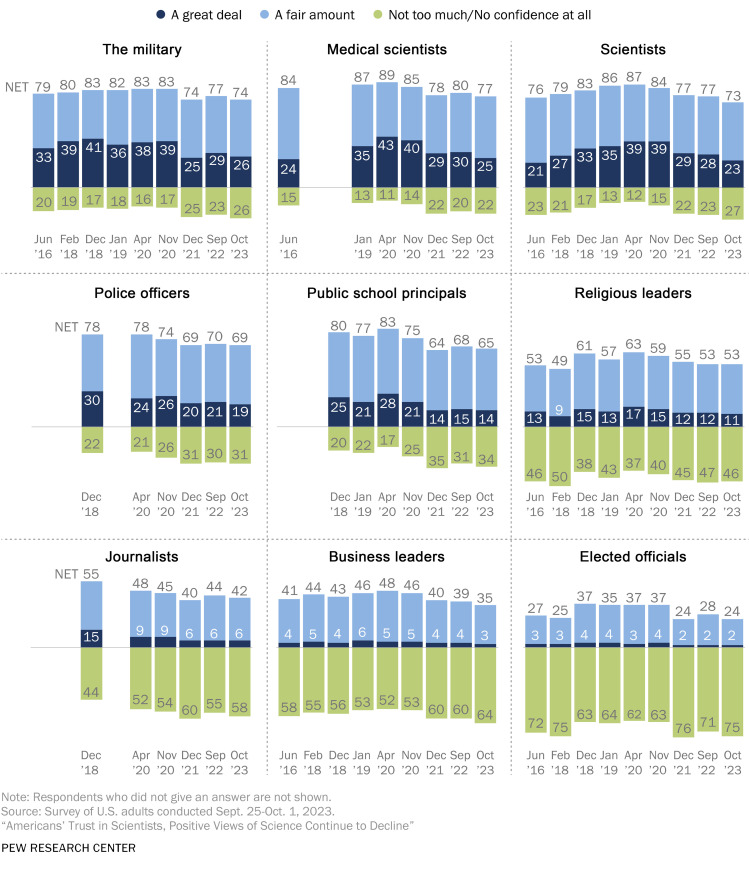
Trust in medical science compared with other areas. Data from the Pew Research Center, 2023. Reproduced from ref. 13.

Calls for restoring Gold Standard science are said to be motivated by the erosion of public trust in science. Although there has been some recent decline in public trust in scientists and medical scientists, especially since the COVID-19 pandemic, it is helpful to look at these data in context (Figure 2) (13). This shift in public trust in scientists should be more fairly viewed against a backdrop of declining trust in many professional areas (notably police officers, the military, public school principals, and journalists). Compared to other areas, public trust in medical scientists remains very high, although we should never take this for granted. We encourage the NIH to include journals and publishers in the conversations to enhance R&R.

Publishing science is a critical component of disseminating research findings, including research funded by NIH. There is currently strong interest in reducing article processing charges. As editors of entirely self-published journals, we can detail the costs required to support the peer review and editorial review processes, data integrity scans, data maintenance, and publishing costs for US-based staffing (14). Publishing gold standard science, like conducting gold standard science, is placed at risk by insufficient funding. Scientific publishing has expanded significantly in the recent decades, producing a wide range of journal quality (15). Ideally, the NIH would ensure that allowable funds for publication costs are spent at journals that require such funds in order to meet high data reporting and reviewing standards, leading to publication of rigorous studies. The scientific community would benefit from knowing which journals follow best practices for transparency in data reporting and data integrity. The NIH and National Library of Medicine are well positioned to provide this information.

Editors, reviewers, NIH-supported scientists, and the scientific community will continue to work with the NIH to ensure the very best use of the public investment in the mission of the NIH, which is to seek fundamental knowledge about the nature and behavior of living systems and the application of that knowledge to enhance health, lengthen life, and reduce illness and disability. This is a noble and worthy cause for all of us to unite in advancing together.

## Version 1 11/03/2025

Electronic publication
